# Construction and Characterization of a Novel Direct Electron Transfer Type Enzymatic Sensor Using Spermidine Dehydrogenase

**DOI:** 10.3390/bios15100681

**Published:** 2025-10-09

**Authors:** Sheng Tong, Yuki Yaegashi, Mao Fukushi, Takumi Yanase, Junko Okuda-Shimazaki, Ryutaro Asano, Kazunori Ikebukuro, Madoka Nagata, Koji Sode, Wakako Tsugawa

**Affiliations:** 1 Department of Biotechnology and Life Science, Graduate School of Engineering, Tokyo University of Agriculture and Technology, 2-24-16 Naka-cho, Koganei 184-8588, Tokyo, Japanmao632332@st.go.tuat.ac.jp (M.F.); yanase.01210@gmail.com (T.Y.); junkoshimazaki@go.tuat.ac.jp (J.O.-S.); ryutaroa@cc.tuat.ac.jp (R.A.); ikebu@cc.tuat.ac.jp (K.I.); 2 Lampe Joint Department of Biomedical Engineering, University of North Carolina at Chapel Hill and North Carolina State University, Chapel Hill, NC 27599, USA; mnagata@email.unc.edu

**Keywords:** direct electron transfer, spermidine dehydrogenase, heme *b*, spermine, pancreatic cancer, chronoamperometry, enzymatic sensor

## Abstract

This study reports on the direct electron transfer (DET) ability of the enzyme spermidine dehydrogenase (SpDH) and its use in a DET-type enzymatic sensor for detecting spermine. SpDH was found to exhibit internal electron transfer from its cofactor, flavin adenine dinucleotide (FAD), to heme *b*. This was confirmed by observing the heme *b*-derived reduction peak at 560 nm in the presence of spermine, the substrate. SpDH was immobilized on a gold electrode via a dithiobis (succinimidyl hexanoate) self-assembled monolayer. The cyclic voltammetry analysis of the SpDH-immobilized gold electrode revealed an increased oxidation current in the presence of 0.1 mM spermine with an onset potential of −0.14 V vs. Ag/AgCl in the absence of an additional external electron acceptor. This result confirmed that SpDH is capable of DET. Chronoamperometric analyses were conducted using an SpDH-immobilized gold electrode with spermine as the substrate under a 0 V oxidation potential vs. Ag/AgCl using an artificial saliva matrix containing 10 µM ascorbic acid and 100 µM uric acid. The sensor exhibited good linear correlation between the current increase and spermine concentration from 0.2 to 2.0 µM, with a limit of detection of 0.084 µM, which encompasses the physiologically relevant spermine concentration found in the saliva. Primary structure alignments and 3D structure predictions revealed that all SpDH homologs possess two conserved histidine residues in the same location on the surface as the heme *b* ligand of SpDH. This indicates their potential for DET-ability with an electrode.

## 1. Introduction

Electrochemical enzymatic biosensors are categorized into three generations based on the electron acceptors used in the oxidative half reaction of the oxidoreductases used in the sensors [1,2]. The first generation uses oxygen as the electron acceptor; the second generation uses artificial electron acceptors; and the third generation uses no external electron acceptor. It is capable of transferring electrons directly to the electrode due to the presence of a “built-in” electron acceptor in its molecule [3,4].

The third-generation principle enzymatic sensor, which uses enzymes capable of direct electron transfer (DET), is considered the ideal design for an enzymatic biosensor, particularly for in vivo continuous monitoring systems. This is due to the following advantages: First, no external synthetic electron acceptor or oxygen is required for sensing. Second, target molecules can be monitored by applying a lower potential, which reduces the impact of electrochemically active ingredients. Third, the sensor structure is simplified.

However, only a limited number of oxidoreductases demonstrated DET with electrodes when immobilized. This is mainly because their redox cofactors—the primary electron acceptors of the reductive half reaction—such as flavin adenine dinucleotide (FAD) and flavin mononucleotide (FMN), are deeply buried in the protein molecule. Consequently, they are too far away to transfer electrons to the electrode when considering the thermodynamic and kinetic diagram of electron transfer between the cofactor and electrode based on Marcus’s theory [3,4,5,6], Among the various cofactor-binding oxidoreductases, some dehydrogenases have an electron transfer subunit or domain that specifically accepts electrons from the redox cofactor in the catalytic site and transfers them to an external electron acceptor. These “built-in mediator” harboring oxidoreductases are reported as DET-type enzymes, which can transfer electrons directly to the electrode. DET-type enzymes can be characterized by the type of redox cofactor or the type of built-in mediator. DET-type enzymes are also divided into two groups: oligomeric enzymes with an electron transfer subunit and monomeric enzymes with an electron transfer domain. A representative example of a DET-type enzyme with an oligomeric structure is the bacterial-derived FAD-dependent glucose dehydrogenase, which contains an electron transfer subunit with three heme *c* molecules [3,4,7]. In contrast, a representative example of a monomeric DET-type enzyme is the quinohemoprotein ethanol dehydrogenase, which contains an electron transfer domain with a heme *c* molecule [2,8].

Additionally, the unique structure of a novel flavocytochrome protein, spermidine dehydrogenase (SpDH; EC1.5.99.6) derived from *Pseudomonas aeruginosa*, PaSpDH, was reported [9]. The enzyme and its homolog derived from *Serratia marcescens* were reported to contain heme and can oxidize spermidine into 1,3-diaminopropane and D1-pyrroline when an electron acceptor such as cytochrome *c*, potassium ferricyanide, or 2,6-dichlorophenolindophenol, is present [10,11,12]. The crystal structure of PaSpDH reveals the presence of heme *b* and FAD as the enzyme’s co-factors. FAD is located in the center of the active site, where it can oxidize the polyamines, such as spermine and spermidine by recognizing the C-N bond of the substrate and transforming itself into a reduced state. It was expected that electrons would transfer from the reduced FAD to heme *b* via intramolecular electron transfer. Most remarkably, the heme *b* of PaSpDH is exposed on the surface of SpDH protein. This unique structural feature inspired our investigation of SpDH’s DET-ability and the subsequent development of a novel DET-type enzymatic sensor using SpDH.

**Scheme 1:**
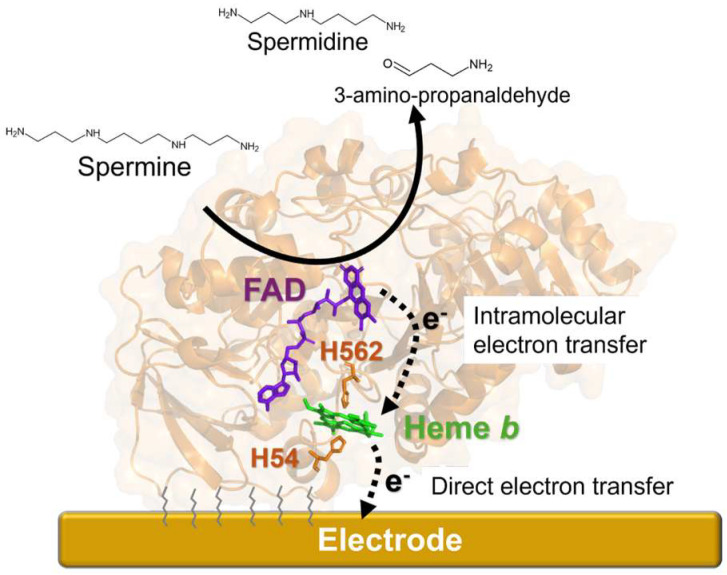
The enzymatic reaction catalyzed by SpDH, and its expected DET with the electrode, to construct a third-generation spermine enzymatic sensor. SpDH (PDB ID: 7D9G) is shown in ribbon representation. Solid arrow indicates the reaction of spermine oxidation catalyzed by SpDH. Dashed arrows indicate the electron transfer from FAD to the electrode via heme *b*. Purple: flavin adenine dinucleotide (FAD), green: heme *b*, brown: axial ligands of heme *b*, histidine 562, and histidine 54 residues.

In this study, we demonstrate the DET-ability of SpDH and construct a third-generation spermine enzymatic sensor (see Scheme 1). Recently, spermine levels in saliva have been recognized as a novel biomarker for accurately diagnosing pancreatic cancer [13]. Considering that the current saliva spermine measurements depend heavily on mass spectrometry [13,14,15,16,17,18] and require complicated equipment operated by professionals, a simple and rapid instrument for monitoring saliva spermine will simplify pancreatic cancer screening using the non-invasive saliva sample. In this study, we used a PaSpDH mutant that had its N-terminal 33 amino acid residues truncated, ΔN33. This mutant has been reported to show higher enzymatic activity than the wild-type enzyme [9]. We first investigated the internal electron transfer between flavin adenine dinucleotide (FAD) and heme *b* using spectrophotometry. This revealed that heme *b* is the primary electron acceptor of reduced FAD during spermine oxidation in SpDH, as evidenced by the observation of a heme *b*-specific reduction peak at 560 nm. An electrochemical investigation using a gold electrode immobilized with PaSpDH, which was covalently attached to a self-assembled monolayer, reveals this enzyme’s DET ability. Consequently, a third-generation principle-based enzymatic spermine sensor was constructed and operated using chronoamperometry with an artificial saliva matrix containing 10 µM ascorbic acid and 100 µM uric acid for monitoring. The sensor exhibited good linear correlation between the current increase and spermine concentration from 0.2 to 2.0 µM, with a limit of detection (LOD) of 0.084 µM, covering the physiologically relevant spermine concentration in saliva. This study is the first to demonstrate that SpDH is capable of DET.

## 2. Materials and Methods

Sodium chloride (NaCl), tris(hydroxymethyl)aminomethane (Tris), imidazole, glycerol, isopropyl β-D-thiogalactopyranoside (IPTG), 5-aminolevulinic acid hydrochloride (5-ALA), iron (III) chloride (FeCl_3_), hydrochloric acid, potassium ferricyanide, sodium dithionite, phenazine methosulfate (PMS), 2,6-dichlorophenolindophenol (DCIP), sodium hydroxide (NaOH) were purchased from Kanto Chemical Co., Inc (Tokyo, Japan). Dithiobis (succinimidyl hexanoate), DSH was purchased from DOJINDO (Kumamoto, Japan). Pt wire was purchased from TANAKA KIKINZOKU (Tokyo, Japan). The Au electrode, and Ag/AgCl reference electrode were purchased from BAS Inc. (Tokyo, Japan).

The recombinant PaSpDH truncated mutant without the N-terminal 33 amino acids PaSpDH (ΔN33) was prepared using *Escherichia coli* (*E. coli*) BL21 (DE3). The detailed methods for recombinant production and purification were described in the Supplementary Materials, Supplementary Methods S1 and S2. The characteristics of the recombinant PaSpDH (ΔN33) are described in the Supplementary Materials, Supplementary Table S1 (Comparison of electron acceptors for PaSpDH), Supplementary Figure S1 (SDS-PAGE analysis of the purified PaSpDH), Figure S2 (Dehydrogenase activities of purified PaSpdH), Figure S3 (Thermal stability of PaSpDH), and Figure S4 (pH dependency of enzymatic activity of PaSpDH).

The enzymatic activity of PaSpDH was determined using PMS/DCIP as the electron acceptors (the PMS/DCIP system). For the PMS/DICP system, the activity measurement was conducted in a phosphate-buffered saline (pH 7.4) with PMS (final concentration [f.c.] of 0.6 mM) as the primary electron acceptor, by monitoring the absorbance change at 600 nm, which corresponds to the peak absorption wavelength of the oxidized DCIP (f.c. of 0.06 mM). Activity was determined by monitoring the decrease in DCIP absorbance at 600 nm (16.3 mM cm^−1^ at pH 7.0). One unit of the enzyme activity is defined as the amount of enzyme that catalyzes the reduction of 1 µmol of DCIP per minute at 25 °C.

Absorption spectra changes in PaSpDH were also observed in the presence of substrate to evaluate the internal electron transfer. The oxidized form of PaSpDH was prepared by incubating 0.1 mM of the purified enzyme with 1 mM of potassium ferricyanide. Then, the enzyme was dialyzed against a 20 mM Tris-HCl buffer solution at pH 8.0. Then, 1 mM spermine was added to the enzyme solution, and the absorbance spectrum was recorded 5 min after adding the substrate. A reduced sample of PaSpDH was prepared by adding 1 mM sodium dithionite. The oxidized, spermine added, and reduced PaSpDH samples were analyzed using a spectrophotometer (V-630 Spectrophotometer, JASCO, Tokyo, Japan).

All electrochemical investigations were carried out using a VSP Electrochemical Measurement System (Bio-Logic Science Instruments, Seyssinet-Pariset, France).

The enzyme electrodes were fabricated using self-assembled monolayer (SAM)-modified electrodes, as previously described [19], with slight modifications. Gold (Au) electrodes (with an electrode surface area of 7 mm^2^) were polished with an alumina and diamond slurry and electrochemically cleaned with 0.5 M NaOH. To construct the SAM-modified electrodes, the washed Au electrodes were immersed overnight in a 100 μL solution of 100 μM DSH at 25 °C. After washing with acetone, the SAM-modified Au electrodes were immersed in 100 μL of a 0.1 μM enzyme solution at 4 °C overnight. The completed enzyme electrodes were stored in 100 mM potassium phosphate buffer (pH 7.0) at 4 °C until use.

Cyclic voltammetry (CV) measurements were conducted using the enzyme-immobilized electrode prepared above as the working electrode, a platinum wire as the counter electrode, and an Ag/AgCl electrode as the reference electrode. Measurements were taken in 100 mM P.P.B. (pH 7.0), with or without 0.1 mM spermine. The potential sweep range and scan rate were set to −0.4 V to 0.4 V vs. Ag/AgCl and 20 mV s^−1^.

Chronoamperometry (CA) measurement was carried out with the same electrode configuration as the CV investigation, by the successive addition of the substrate solution (0.025–100 µM spermine) in modified Fusayama artificial saliva (artificial saliva), which consisted of 5 mM Na_2_HPO_4_, 5.4 mM CaCl_2_, 5.4 mM KCl, 6.8 mM NaCl, 4 g/L mucin from porcine stomach, 66 mM urea, 100 μM uric acid, and 10 μM ascorbic acid (pH 7.2) [20] at 25 °C, under the applied potential of 0 mV vs. Ag/AgCl. For calibration, the average of twenty values acquired over a duration of 10 s prior to the addition of the next substrate was used.

We evaluated the impact of ingredients on the sensor response using CA with an enzymatic electrode, an Ag/AgCl electrode, and a Pt wire as the working electrode, the reference electrode, and the counter electrode, respectively. The electrodes were immersed in 2 mL of the basal artificial saliva solution which was the modified Fusayama artificial saliva without urea, uric acid, and ascorbic acid, under stirring by a magnetic stirrer. A potential of 0 V (vs. Ag/AgCl) was then applied. The current was recorded as the interference solution was added sequentially (final concentrations of 10 and 100 μM ascorbic acid, 10 and 100 μM uric acid, and 66 mM urea). After adding all the interferences, 100 μM of spermine was added to confirm the activity of the enzyme on the working electrode.

Homology searches were performed using the NCBI BLASTP web server (https://blast.ncbi.nlm.nih.gov/) (accessed 15 April 2025).

## 3. Results and Discussion

### 3.1. Intramolecular Electron Transfer in PaSpDH

**Figure 1:**
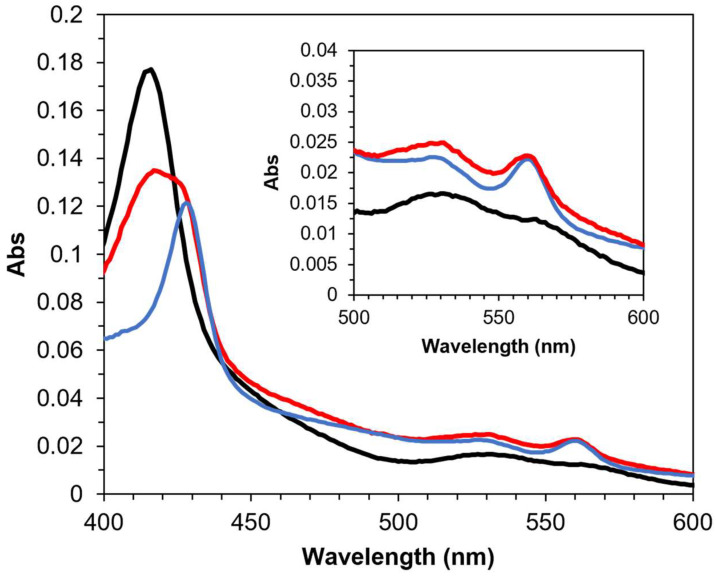
Spectroscopic analyses of PaSpDH ΔN33. Black line: the oxidized state. Red line: the reduction state. Blue line: immediately after the addition of 1 mM spermine to the oxidized enzyme sample.

Based on structural investigations, the primary electron acceptor of FAD in PaSpDH was postulated to be heme *b*. However, no investigation about intra-molecular electron transfer has been reported for this enzyme. Therefore, we examined the spectroscopic observation of the intramolecular electron transfer between FAD and heme *b* by observing the reduction spectrum of heme *b* during the enzymatic reaction. The oxidized state of PaSpDH was prepared by incubating an enzyme sample with the synthetic electron acceptor ferricyanide, which exhibited a Soret peak at approximately 415 nm, corresponding to heme (Figure 1). Reducing the enzyme with 1 mM sodium dithionite resulted in a clear increase in peaks at 530 and 560 nm. These peaks correspond to reduced heme *b*, indicating that heme *b* reduction occurred. Adding 1 mM spermine to the fully oxidized enzyme sample immediately increased the peaks at 530 and 560 nm, confirming heme *b* reduction. These results suggest that electrons are transferred from FAD to heme *b* via intramolecular electron transfer. This confirms that heme *b* is the primary electron acceptor of FAD in the PaSpDH reaction.

### 3.2. Investigation of DET-Ability of PaSpDH

**Figure 2:**
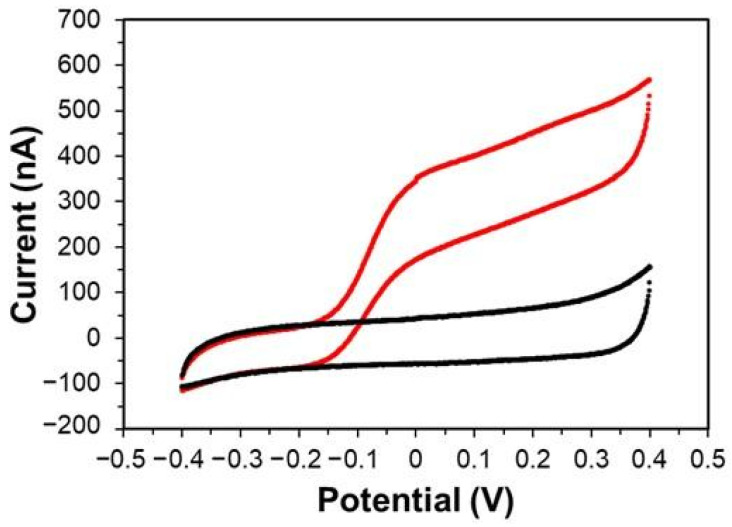
CV measurement of PaSpDH-modified Au electrode with or without spermine. Black: without spermine, Red: with 0.1 mM spermine addition, under Ar atmosphere.

PaSpDH was immobilized on DSH-SAM-modified gold disk electrodes via amine coupling to perform electrochemical measurements. First, cyclic voltammetry (CV) measurement was carried out in the presence and absence of spermine without the use of synthetic electron acceptors (Figure 2). For the baseline control, a CV measurement was also carried out using an electrode without an enzyme (see Supplementary Materials, Figure S5). When spermine was added in the solution, a clear PaSpDH catalytic current was observed. This indicates PaSpDH’s DET ability. Considering that the redox cofactor, FAD, is deeply buried in the protein molecule, heme *b* is probably responsible for this DET ability. The onset potential was also observed to be approximately −0.14 V vs. Ag/AgCl. This result also indicates that the third-generation-based enzymatic spermine sensor can operate at lower potentials, avoiding the impact of the potential electrochemical interferents. To the best of our knowledge, this is the first unveiled structure of a monomeric oxidoreductases with a DET-ability that contains both flavin- and heme-binding modules without a connecting linker region. Only an oxidation current was observed from these CVs in the presence of spermine (Figure 2). In the absence of the substrate, the CV was identical to that of the electrode without the enzyme (see Figure S5), and no oxidation or reduction peak was observed. These observations were consistent with that previously reported in other DET-type enzyme-based CV investigation, using FAD-dependent glucose dehydrogenases from *Burkhorderia cepacia* and fructose dehydrogenase from *Gluconobacter japonicus* [4].

### 3.3. Characterization of the Third-Generation-Type Spermine Sensor

**Figure 3:**
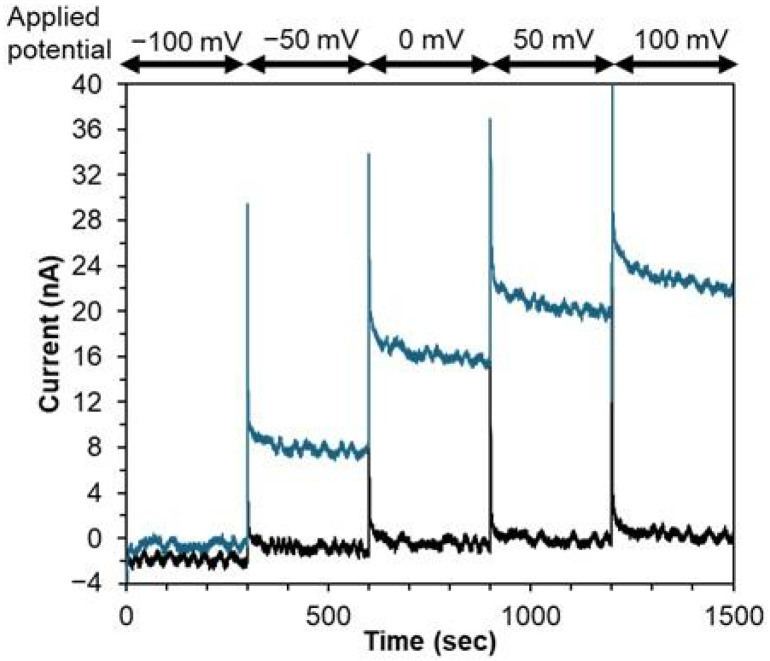
Effect of applied potential on the sensor response. Blue: w/1 µM spermine, Black: w/o spermine. Applied potential: −100, −50, 0, 50, and 100 mV vs. Ag/AgCl.

We investigated the third-generation-type enzymatic sensor for spermine using a PaSpDH-immobilized gold electrode with chronoamperometric modality in artificial saliva to demonstrate its feasibility for future clinical applications. Figure 3, shows the sensor response dependency on the oxidation potential. The sensor signal can be observed from −0.05 V vs. Ag/AgCl, which is a significant advantage of this new DET-type enzymatic sensor using SpDH. Considering that the observed current intensity is dependent on the applied potential and background noise level, we deemed 0 V vs. Ag/AgCl as the applied potential for the further CA measurement, and we can avoid the direct oxidation of acetaminophen and ascorbic acid.

**Figure 4:**
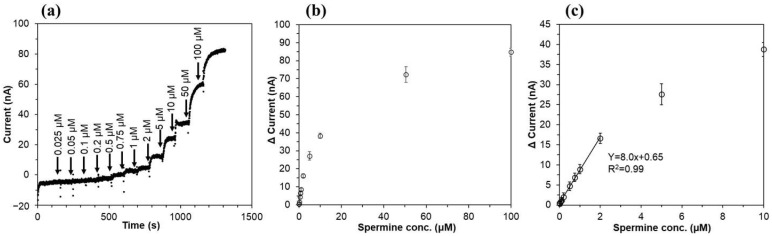
Chronoamperometric measurement of spermine: (**a**) Representative amperometric sensor response, (**b**) calibration curve of spermine sensor in triplicate, plotting the current of full concentration range, and (**c**) low (<10 μM) concentration range.

Figure 4a shows the amperometoric sensor response, Figure 4b,c shows the correlations between the current increase and spermine concentrations. As the spermine concentration increased, a clear increase in current was observed from 0.1 µM to 100 µM spermine concentration under a potential of 0 V vs. Ag/AgCl. The response time is defined as the time to reach ±5% of the steady-state signal for the calibration curve. We used the current values which were observed after 110 s of sample injection and averaged twenty values acquired over a duration of 10 s prior to the addition of the next substrate to prepare the calibration curves in this study. The detailed sensor CA response towards 1 µM spermine, which corresponds to a healthy subjects’ spermine concentration in saliva, was shown in the Supplementary Materials, Figure S6. The sensor exhibited good linear correlation between the current increase and spermine concentration from 0.2 to 2.0 µM, y = 8.0x + 0.65 (R^2^ = 0.99), with a limit of detection (LOD) of 0.084 µM, covering the physiologically relevant spermine concentration in saliva. Since the salivary spermine concentration of pancreatic cancer patients is between 1.5 and 8.5 µM, these results suggest that the sensor can monitor physiologically relevant spermine concentrations in saliva (Figure 4c). The relative standard deviation (RSD) was 6% for same electrode repeatability (*n* = 3) and 13% for inter-electrode reproducibility (*n* = 3). The high variability among different electrodes is likely due to the lack of standardization in the fabrication process, which can be improved with a more controlled construction method.

**Figure 5:**
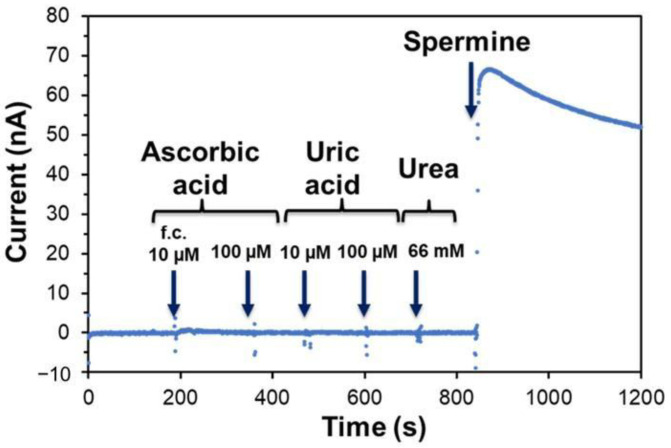
Investigation of the impact of the addition of ingredients on the sensor response. The electrochemically active ingredients, ascorbic acid (10 and 100 μM), uric acid (10 and 100 μM), and urea (66 mM) were subsequently injected in 2 mL of the basal artificial saliva solution, which was the modified Fusayama artificial saliva without urea, uric acid, and ascorbic acid, under stirring by a magnetic stirrer, under 0 mV vs. Ag/AgCl was applied to the enzymatic sensor. After adding all the interferences, 100 μM of spermine was added to confirm the activity of the enzyme on the working electrode.

To investigate the potential interferents in saliva for monitoring, we examined the impact of ascorbic acid and uric acid in the basal artificial saliva components that did not contain urea, uric acid, or ascorbic acid (Figure 5). Adding 10 µM or 100 µM of ascorbic acid, or uric acid, and 66 mM urea to the basal artificial saliva did not cause any significant baseline change. However, adding spermine to the mixture clearly increased the current in the chronoamperometric investigation. These results suggest that the enzymatic sensor using SpDH is unaffected by potential interferents that may be present in the saliva or other biological samples.

To compare the DET-type and MET-type sensors using SpDH, CA measurements were carried out using 6 mM methoxy-phenazinemethosulphate (mPMS) as a mediator at 0.2 V vs. Ag/AgCl, which is shown in Supplementary Materials, Figure S7. The time course showed the increase in spermine concentration-dependent currents (Figure S7a). The sensor showed about 100 nA and 300 nA toward 10 µM and 300 µM spermine, respectively (Figure S7b). This is an approximately five-fold higher catalytic current compared with DET-type enzymatic sensor mode at 0 V vs. Ag/AgCl in the absence of a mediator (and 20 nA and 60 nA toward 10 µM and 300 µM spermine, respectively). These results indicated that MET-type sensor modality with SpDH showed a higher current compared with DET-type modality. However, in the absence of a mediator for measurements in varieties of conditions, not only for in vivo continuous monitoring, but also for disposable sensor for POCT usage, DET-type modality provides significant advantages, such as not having to consider cytotoxicity and the leakage of mediators for in vivo use, the chemical instability of mediators, and cost effectiveness due to the absence of mediators as the sensor constituents.

This article demonstrated that a novel flavocytochrome protein, PaSpDH, which is an FAD oxidoreductase harboring heme *b*, is a DET-type enzyme, and can be used in a third-generation principle-based enzymatic sensor. Pioneering work by Che et al. [9] reported the X-ray crystal structure of PaSpDH, revealing its unique dehydrogenase structure. Several oxidoreductases with DET-abilities have been reported, which have monomeric enzyme structures with an electron transfer domain. The electron transfer domains are clearly distinct from the catalytic domains and are connected to them via a linker region. The catalytic domains harbor the catalytic sites and primary electron acceptors (FAD, FMN, and PQQ). Among the GMC oxidoreductase family, the best-known enzyme with this structure is the FAD-dependent cellobiose dehydrogenase [2,4,21,22,23,24]. The FAD-harboring catalytic domain is connected with the heme *b*-harboring electron transfer domain and shows DET with an electrode. The unique location of heme *b* is on the surface of the enzyme and is chelated by two histidine residues, His54 and His562. Che et al. also reported on the presence of these two characteristics and conserved His residues in the previously reported SpDH derived from *Citrobacter freundii*, *Serratia marcescens*, and their homologues, *Yersinia ruckeri*, *Neisseria weaveri*, and *Cupriavidus gilardii*, showing the primary structure alignment at the N-terminal region with His54 residues. To expand our understanding, we analyzed the entire primary structure alignments of these four SpDHs, which were referenced by Che et al. [9], and predicted their 3D structures using AlphaFold3. The primary structure alignments clearly demonstrate that they all possess two conserved histidine residues (see Supplementary Information, Figure S8). As expected from the primary structure alignment, these two His residues exist in the same location as in PaSpDH. They exactly overlap with PaSpDH His54 and His562, thereby possibly harboring heme *b* in the same position as PaSpDH (see Supplementary Information, Figure S8). We then used BLAST protein analyses to search for homologues of PaSpDH and anonymously selected two homologues that cover 99% or more of the sequence with primary structure identities of less than 50%, namely *Cupriavidus necator*-derived putative proteins (45.5%) and *Candidatus Poseidoniales archaeon*-derived putative proteins (41.5%). These two homologues were revealed to harbor two conserved His residues in the same positions as PaSpDH. Their AlphaFold3-predicted structures indicate that they may retain heme *b*, which is chelated by two conserved His residues. However, their substrates are not known (see Supplementary Information, Figure S9a–g). Considering that PaSpDH showed a DET ability with an electrode due to the presence of a unique heme *b* on the enzyme’s surface, chelated by two His residues as the primary electron acceptor of FAD, all of these homologues will also show DET ability with an electrode. They will be able to serve as unique biological recognition elements for third-generation electrochemical enzymatic sensors when their cognate substrates are identified.

Recently, the detection of spermine as a cancer biomarker has presented significant analytical challenges requiring high sensitivity and selectivity. Biological samples, such as saliva, urine, and blood, contain numerous interfering compounds, while spermine concentrations are typically very low (μM range). These factors necessitate exceptional performance characteristics in biosensors. Additionally, cancer screening requires the processing of large sample volumes, necessitating cost-effective, user-friendly, and rapid detection methods. While accurate, conventional analytical techniques such as LCMS and GCMS are impractical for widespread screening due to their complexity and resource requirements. Given these constraints, there is an urgent need for an accessible, highly sensitive spermine biosensor. We propose that electrochemical sensing technology is the ideal solution because it combines the necessary analytical performance with practical applicability for large-scale screening programs.

Table 1 provides a comprehensive comparison of electrochemical spermine sensors. Among these, Zaman et al. demonstrated exceptional sensitivity with an Fe/MIPpy nanozyme, achieving a limit of detection (LOD) of 220 pM [25]. Although other groups, including ours, have reported detection ranges in the nanomolar to the micromolar range, these sensitivities are adequate for monitoring spermine in the physiological range because sub-micromolar sensitivity is typically sufficient for clinical applications. Our sensor offers distinct advantages over existing approaches. Unlike conventional sensors, which require mediators such as ferricyanide, Prussian blue, or hydrogen peroxide, our system uses a DET-type enzyme to enable detection at 0 V vs. Ag/AgCl. This mediator-free approach solves two critical issues. First, detecting hydrogen peroxide or several mediators requires a higher overpotential, which can interfere with electroactive species, such as ascorbic acid. Second, their sensitivity to environmental factors, such as light and pH, compromises sensor stability during storage and practical applications. Since the enzymatic sensor stability is strongly dependent on the stability of the enzyme itself, we investigated the thermal stability of SpDH (Supplementary Materials, Figure S3a,b). SpDH is at a stable temperature below 40 °C, which is recognized as a moderately stable enzyme. Although further investigations are necessary to address storage stability and long-term operational stability, this moderate stability of SpDH indicates the feasibility in the use of the disposable enzyme sensor strip. Investigating SpDH using a third-generation enzymatic sensor revealed that the sensor can monitor spermine in the salivary concentration range in an artificial saliva sample containing electrochemically active ingredients such as 10 µM ascorbic acid and 100 µM uric acid.

Furthermore, it simplifies sensor design and reduces operational complexity, making it ideal for point-of-care testing and large-scale, spermine-based cancer screening programs for patients. While our sensor effectively addresses the challenges of stability, interference, and operational complexity through its DET capability, making it a promising candidate for the future spermine sensor, it still faces limitations in selectivity with spermidine. This stems from the enzyme’s inherent characteristics. However, we anticipate that the targeted biomolecular engineering of the SpDH will resolve this selectivity issue and further enhance the sensor’s practical utility.

## 4. Conclusions

The numbers and variation in enzymes capable of DET ability with electrodes are limited, so finding and demonstrating a new DET-enzyme is valuable for biosensor development in the biomedical field. In this study, we investigated the DET ability of SpDH, which harbors the FAD and surface-exposed heme *b*, and the subsequent development of a novel DET-type enzymatic sensor using SpDH. SpDH was found to exhibit internal electron transfer from its cofactor, FAD, to heme *b*. The cyclic voltammetry analysis of the SpDH-immobilized gold electrode revealed an increased oxidation current in the presence of 0.1 mM spermine with an onset potential of −0.14 V vs. Ag/AgCl in the absence of an additional external electron acceptor. This result confirmed that SpDH is capable of DET. Chronoamperometric analyses exhibited good linear correlation between the current increase and spermine concentration from 0.2 to 2.0 µM, with a limit of detection of 0.084 µM. The electrochemically active ingredients such as 10 µM ascorbic acid and 100 µM uric acid do not influence the spermine detection. These results demonstrated that the DET-type enzymatic sensor using SpDH is a promising candidate for a future spermine sensor.

In addition, this is the first monomeric oxidoreductase with a DET-ability that contains both flavin- and heme-binding modules without a connecting linker region. Primary structure alignments and 3D structure predictions for SpDH homologs revealed that all of the homologs possess two conserved histidine residues in the same location as SpDH. These residues may serve as heme *b* ligands, indicating the homologs’ potential for their DET-ability with an electrode. Once their cognate substrates are identified, these homologs can serve as unique biological recognition elements for third-generation electrochemical enzymatic sensors.

## Figures and Tables

**Table 1:** Comparison of electrochemical biosensor for spermine detection.

Recognition Element	Detection Principle	Target	Sensitivity	Selectivity	Sample Type	Reference
PAO ^1^ enzyme	Amperometric H_2_O_2_ detection with Prussian blue (−100 mV vs. Ag/AgCl)	Spermine, spermidine	Linear range: 0.003–0.3 mM (spermine), 0.01–0.4 mM (spermidine)	Selective for spermine/spermidine against other polyamines	Blood	[26]
SMO ^2^ enzyme	Amperometric H_2_O_2_ detection with Prussian blue (−100 mV vs. Ag/AgCl)	Spermine	Linear range: 0.004–0.5 mM	Selective for spermine against other polyamines	Blood	[26]
MIP ^3^ nanofilm	EGFET	Spermine	LOD: 1.23 ng/mL (6.08 nM)	Selective for spermine over spermidine and histamine	Urine	[27]
Fe/MIPpy ^4^ nanozyme	EIS/CV with ferricyanide	Spermine	LOD: 220 pM, LOQ: 667 pM	Selective for spermine over other saliva component	Saliva	[25]
Poly(β-cyclodextrin)	Direct electrochemical oxidation by DPV (0.14 V vs. Ag/AgCl)	Spermine	LOD: 0.001 mg/L (4.94 nM)	Selective for spermine over other blood component	Blood	[28]
SpDH	DET-enzyme amperometry (0 V vs. Ag/AgCl)	Spermine	Linear range: 0.2–2.0 µM LOD: 0.084 µM	Selective for Spermine/spermidine over other artificial saliva component	Artificial saliva	this work

^1^ PAO; polyamine oxidase, ^2^ SMO; spermine oxidase, ^3^ MIP; molecularly imprinted polymer, ^4^ py; polypyrrole.

## Data Availability

The original contributions presented in this study are included in the article/Supplementary Material. Further inquiries can be directed to the corresponding authors.

## Supplementary Materials

The following supporting information can be downloaded at https://www.mdpi.com/article/10.3390/bios15100681/s1. Method S1: Construction of PaSpDH expression vector; Method S2: Preparation of PaSpDH; Table S1: Comparison of the electron acceptors for PaSpDH; Figure S1: SDS-PAGE analysis of the purified PaSpDH; Figure S2: Dehydrogenase activities of purified PaSpDH; Figure S3: Thermal stability of PaSpDH; Figure S4: pH dependency of enzymatic activity of PaSpDH; Figure S5: CV measurement of SAM-modified gold electrode with or without spermine; Figure S6: Response time of the enzymatic sensor; Figure S7: Chronoamperometric measurement of spermine in the presence of electron mediator; Figure S8: Entire primary structure alignment of PaSpDH and its homologs; and Figure S9: Structure of PaSpDH and its homologs predicted by AlphaFold3.
