# Supplementary material for: Construction and Characterization of a Novel Direct Electron Transfer Type Enzymatic Sensor Using Spermidine Dehydrogenase
Source: Biosensors (Basel). 2025 Oct 9;15(10):681. doi: 10.3390/bios15100681 (PMC12564564; doi:10.3390/bios15100681)
Supplement: Supplementary file 1 [file biosensors-15-00681-s001.zip › biosensors-3833173-supplementary.pdf]

biosensors

MDPI

Supplementary Materials

Construction and Characterization of a Novel Direct Electron Transfer Type Enzymatic Sensor Using Spermidine Dehydrogenase

Sheng Tong [1,†], Yuki Yaegashi [1,†], Mao Fukushi [1,†], Takumi Yanase [1], Junko Okuda-Shimazaki [1], Ryutaro Asano [1], Kazunori Ikebukuro [1], Madoka Nagata [2], Koji Sode [2,*] and Wakako Tsugawa [1,*]

[1] Department of Biotechnology and Life Science, Graduate School of Engineering, Tokyo University of Agriculture and Technology, 2-24-16 Naka-cho, Koganei 184-8588, Tokyo, Japan; mao632332@st.go.tuat.ac.jp (M.F.); yanase.01210@gmail.com (T.Y.); junkoshimazaki@go.tuat.ac.jp (J.O.-S.); ryutaroa@cc.tuat.ac.jp (R.A.); ikebu@cc.tuat.ac.jp (K.I.)

[2] Lampe Joint Department of Biomedical Engineering, University of North Carolina at Chapel Hill and North Carolina State University, Chapel Hill, NC 27599, USA; mnagata@email.unc.edu

* Correspondence: ksode@email.unc.edu (K.S.); tsugawa@cc.tuat.ac.jp (W.T.)

† These authors contributed equally to this work.

Table of contents

Supplementary Methods

Method S1. Construction of PaSpDH expression vector.
Method S2. Preparation of PaSpDH.

Supplementary Table and Figures

Table S1. Comparison of the electron acceptors for PaSpDH.
Figure S1. SDS-PAGE analysis of the purified PaSpDH.
Figure S2. Dehydrogenase activities of purified PaSpDH.
Figure S3. Thermal stability of PaSpDH.
Figure S4. pH dependency of enzymatic activity of PaSpDH.
Figure S5. CV measurement of SAM-modified gold electrode with or without spermine.
Figure S6. Response time of the enzymatic sensor.
Figure S7. Chronoamperometric measurement of spermine in the presence of electron mediator.
Figure S8. Entire primary structure alignment of PaSpdH and its homologs.
Figure S9. Structure of PaSpdH and its homologs predicted by AlphaFold3.

 https://doi.org/10.3390/bios15100681

Supplementary Methods

Method S1. Construction of PaSpDH expression vector.

The SpDH derived from *Pseudomonas aeruginosa* PAO1 was used for this study (GenBank accession No. AAG07100). A synthetic *P. aeruginosa* full-length SpDH (PaSpDH) gene optimized for *Escherichia coli* codon usage was obtained from Eurofins Genomics (Tokyo, Japan). This gene was amplified by PCR using a forward primer (5′-AGAAGGAGATATACATATGACCATCTCTCGTCGCGACTTC-3′, containing a *Nde*I restriction site) and a reverse primer including a *Hin*dIII restriction site (5′-TCGAGTGCGGCCGCAAGCTTCCCAAGCTCACGCACGG-3′) and inserted into the multi-cloning site of the expression vector pET30c (+) to construct pET30c-PaSpDH. The PaSpDH mutant, deleting 33 amino acids from the N-terminal of PaSpDH, ΔN33 PaSpDH, was prepared using the pET30c-PaSpDH plasmid as a template. The ΔN33 forward primer sequence was as follows: 5′-AGAAGGAGATATACATATGCGCTACTATCCGCCGGCAC-3′, containing a *Nde*I restriction site. The reverse primer sequence, including a *Hin*dIII restriction site, was as follows: 5′-TCGAGTGCGGCCGCAAGCTTCCCAAGCTCACGCACGG-3′. The PCR product was purified and digested with *Nde*I and *Hin*dIII. Digested PCR products encoding ΔN33 PaSpDH were cloned into the vector pET30c (+) to obtain pET30c-ΔN33 PaSpDH plasmid.

Method S2. Preparation of PaSpDH

E. coli BL21 (DE3) was transformed with pET30c-ΔN33 PaSpDH and grew as a preculture in 3.0 mL Luria–Bertani broth (LB) medium containing 50 μg mL^{-1} kanamycin at 37 °C for 18 h, 140 rpm. A 1.0 mL of preculture was inoculated to 100 mL LB medium containing 50 μg mL^{-1} kanamycin at 37 °C, 170 rpm until OD_{660} = 0.5. Then, IPTG (f. c. 0.3 mM), 1 mM 5-ALA, and 500 μM $FeCl_3$ were added into the culture medium and incubated at 16 °C for 18 h, 170 rpm. After harvesting, the wet cells were resuspended with 3 mL/g wet cell weight in 20 mM Tris-HCl (pH 8.0) containing 0.5 M NaCl and 20 mM imidazole and disrupted by a French press. The resulting cell extracts were centrifuged (10,000 xg) at 4 °C for 20 min to separate the cell debris and supernatant. The supernatant was then ultracentrifuged (106,000 xg) at 4 °C for 30 min, and the crude enzyme was prepared as supernatant. Thus, the prepared crude enzyme was applied to the HisTrap HP column (Cytiva, MA, USA) equilibrated with 20 mM Tris-HCl (pH 8.0) containing 0.5 M NaCl and 20 mM imidazole. Protein was eluted with a linear gradient of imidazole. Active fractions were collected and diluted in 20 mM Tris-HCl (pH 8.0) with 0.1 M NaCl and 10% glycerol. The collected fractions were then examined with SDS-PAGE analysis (Figure S1) and activity assay.

Supplementary Table and Figures

Table S1. Comparison of the electron acceptors for PaSpDH.

	Ru/MTT	MTT	PMS/DCIP	DCIP	$K_3Fe(CN)_6$
Specific activity ($U\ mg^{-1}$)	17	0.14	49	5.2	140

Abbreviations: Ru: Hexaammineruthenium(III) chloride; MTT: Thiazolyl blue tetrazolium bromide; PMS: phenazine methosulfate; DCIP: 2,6-dichlorophenolindophenol.

Activity measurements were conducted with 1 mM spermine in a 20 mM phosphate-buffered saline (pH 7.4) containing each reagent (Ru/MTT system: 77 mM Ru and 1 mM MTT, MTT system: 1 mM MTT, PMS/DCIP system: 0.6 mM PMS and 0.06 mM DCIP, DCIP system: 0.06 mM DCIP, potassium ferricyanide system: 0.5 mM $K_3Fe(CN)_6$. Dehydrogenase activity was measured differently depending on the system used. For Ru/MTT system and MTT system, by monitoring a reduction in MTT at 565 nm. For PMS/DCIP system and DCIP system, dehydrogenase activity was measured by monitoring the reduction in DCIP at 600 nm. For potassium ferricyanide system, dehydrogenase activity was measured by monitoring reduction in $K_3Fe(CN)_6$ at 420 nm. Both the potassium ferricyanide system and PMS/DCIP system showed high activity for PaSpDH. Given that the molecular extinction coefficient of DCIP (16.3 $mM^{-1}\ cm^{-1}$) is significantly higher than that of ferricyanide (1.04 $mM^{-1}\ cm^{-1}$), the PMS/DCIP system was selected for measuring spermine due to its higher sensitivity.

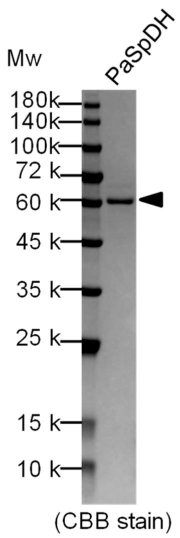

Figure S1. SDS-PAGE analysis of the purified PaSpDH. Left: The molecular weight marker; Right: 5 µg of purified enzyme sample.

The major band (black arrow) corresponding to the expected molecular weight of PaSpDHΔN33 (67 kDa) is shown.

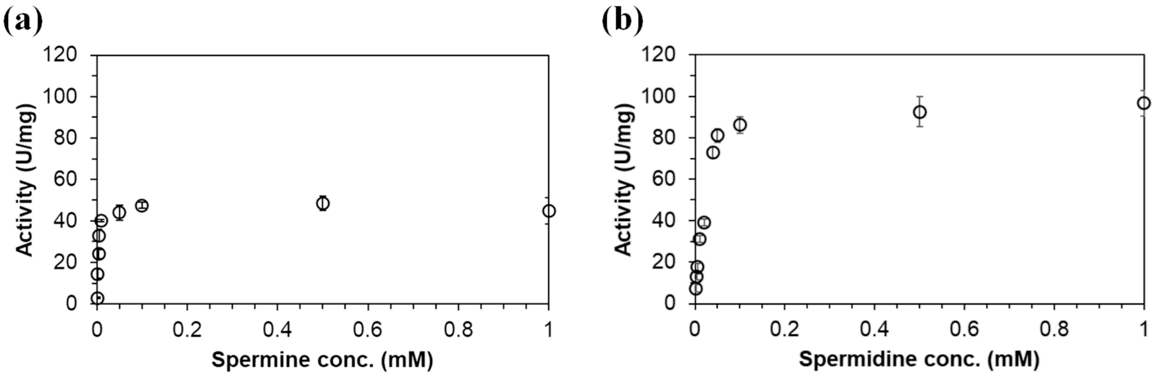

Figure S2. Dehydrogenase activities of purified PaSpDH.

The (**a**) spermine or (**b**) spermidine dependence on the specific activities of PaSpDH. The activity was measured in 20 mM phosphate-buffered saline (pH 7.4) containing 0.06 mM DCIP and 0.6 mM PMS by monitoring the reduction in DCIP at 600 nm.

The Michaelis–Menten kinetic parameters, K_m and V_{max}, were determined from the Hanes–Woolf plots. The initial rates were obtained from each curve of the substrate-dependent activity. ((a) For spermine, K_m: 0.0037 mM, V_{max}: 48 U mg^{-1}; (b) For spermidine, K_m: 0.021 mM, V_{max}: 101 U mg^{-1}).

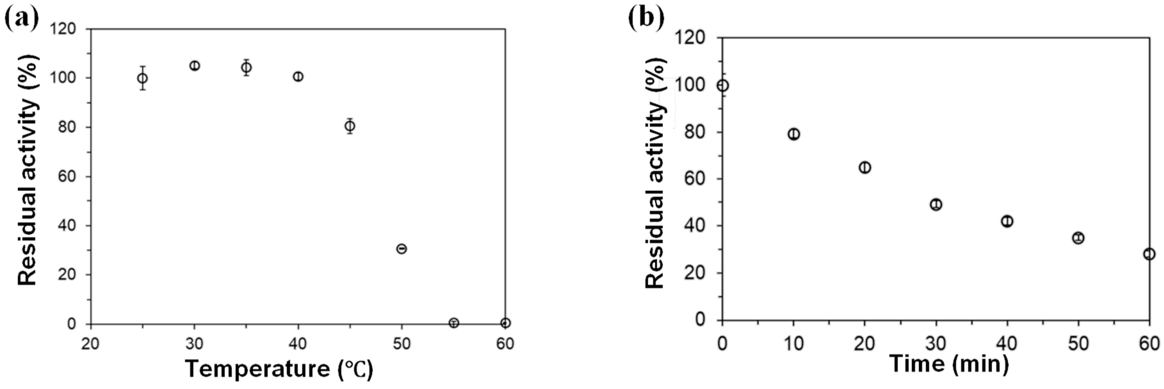

Figure S3. Thermal stability of PaSpDH: (**a**) Temperature-dependent inactivation of PaSpDH; and (**b**) Time course of the activity at 45 °C.

The thermal stability of PaSpDH was evaluated and the residual activity at each temperature was expressed as a percentage of the activity at 25 °C, as 100%. The purified enzyme sample of PaSpDH was used. (a) After incubating the enzyme sample at each temperature (25, 30, 35, 40, 45, 50, 55, 60 °C) for 10 min, the activity toward 1 mM spermine was measured in 20 mM phosphate-buffered saline (pH 7.4) by PMS/DCIP system. The residual activity values were calculated by the activity value measured using the enzyme sample incubated at 25 °C as 100% and plotted against the incubation temperature. (b) After incubating at 45 °C for various times (0, 10, 20, 30, 40, 50, 60 min), the activity was measured in the same way as (a). The residual activity values were calculated by the activity value measured using the enzyme sample incubated for 0 min as 100% and plotted against the incubation time. As shown in (a), the incubation of the enzyme at temperatures above 40 °C for 10 min decreased in activity, and no residual activity was observed at

temperatures of 55 °C or higher. As indicated in (b), prolonged incubation leads to a progressive decline in enzymatic activity.

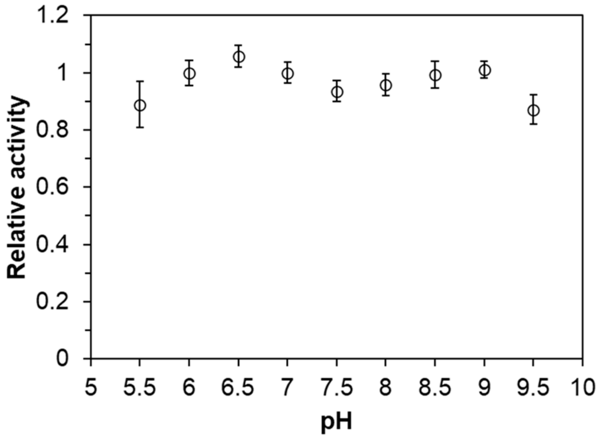

Figure S4. pH dependency of enzymatic activity of PaSpDH.

The pH dependency of the enzymatic activity of PaSpDH was evaluated by comparing it with the activity measured at pH 7.0. The purified enzyme sample was used. The activity toward 1 mM spermine was measured in various buffers (20 mM acetate buffer (pH 5.5), 20 mM P.P.B. (pH 6.0, 6.5, 7.0, 7.5, 8.0), and 20 mM Tris-HCl buffer (pH 8.5, 9.0, 9.5)) by the PMS/DCIP system.

PaSpDH showed no significant difference in activity at pH 5.5–9.5.

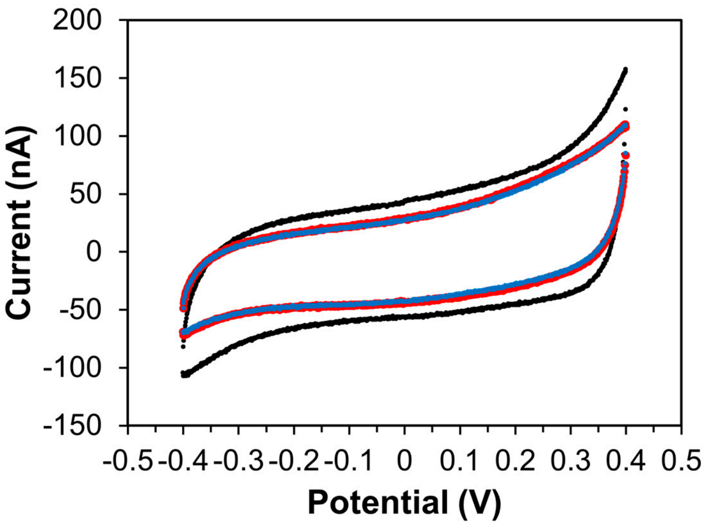

Figure S5. CV measurement of SAM-modified gold electrode with or without spermine.

The CV measurement was performed on a SAM-modified gold electrode under Ar atmosphere to investigate the electrochemical behavior in the presence and absence of 0.1 mM spermine. The blue line represents the baseline measurement of the SAM-modified gold electrode without spermine and without the immobilized enzyme. The red line shows the measurement with 0.1 mM spermine but without the enzyme. The black line represents the CV of PaSpDH-immobilized electrode in the absence of spermine (this trace being identical to the data shown in Figure 2).

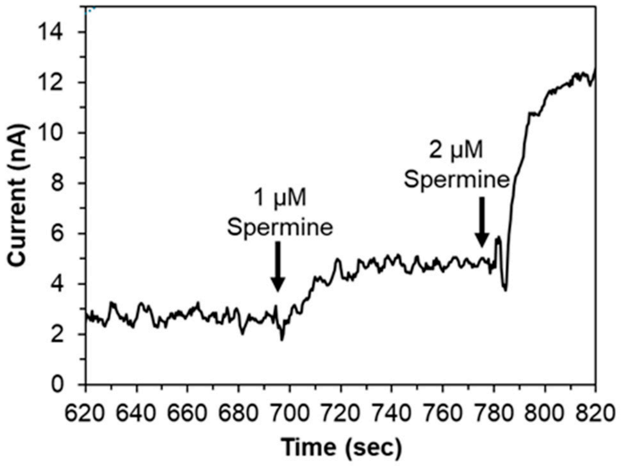

Figure S6. Response time of the enzymatic sensor.

The arrow indicates the addition of 1 and 2 μM of spermine. Enlarged view of the data points from 620 to 820 s in Fig. 4a.

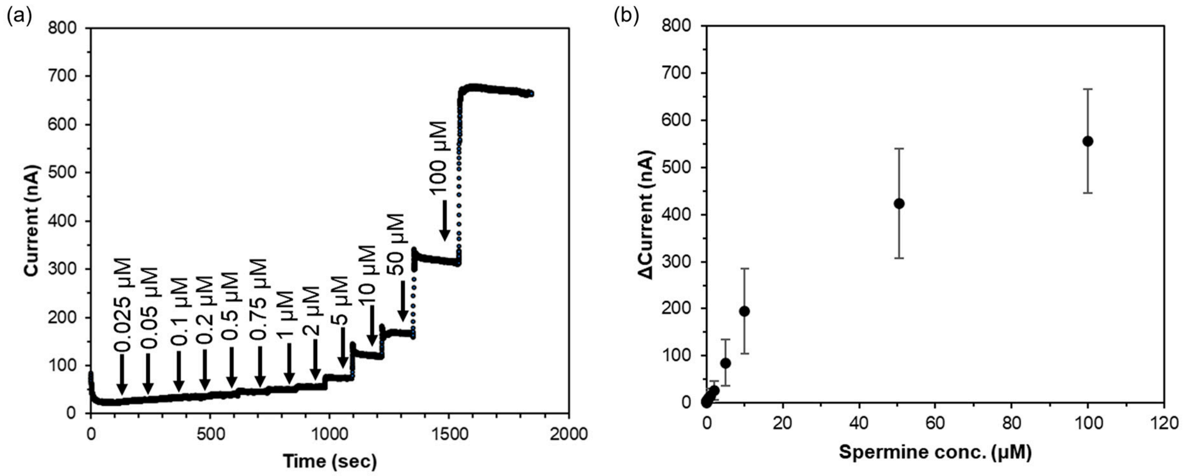

Figure S7. Chronoamperometric measurement of spermine in the presence of electron mediator: (**a**) Representative amperometric sensor response; (**b**) Calibration curve of spermine sensor in triplicate. CA measurements were carried out using PaSpDH-immobilized electrodes in the presence of 6 mM methoxy phenazine methosulfate in 100 mM potassium phosphate buffer (pH 7.0) at room temperature. Spermine was successively added to the test solution (0.025–100 μM spermine). PaSpDH-immobilized gold electrode (3 mmφ) was used as the working electrode. Pt wire and Ag/AgCl were utilized as the counter electrode and reference electrode, respectively. CA measurements were performed at an applied potential of 0.2 V vs. Ag/AgCl.

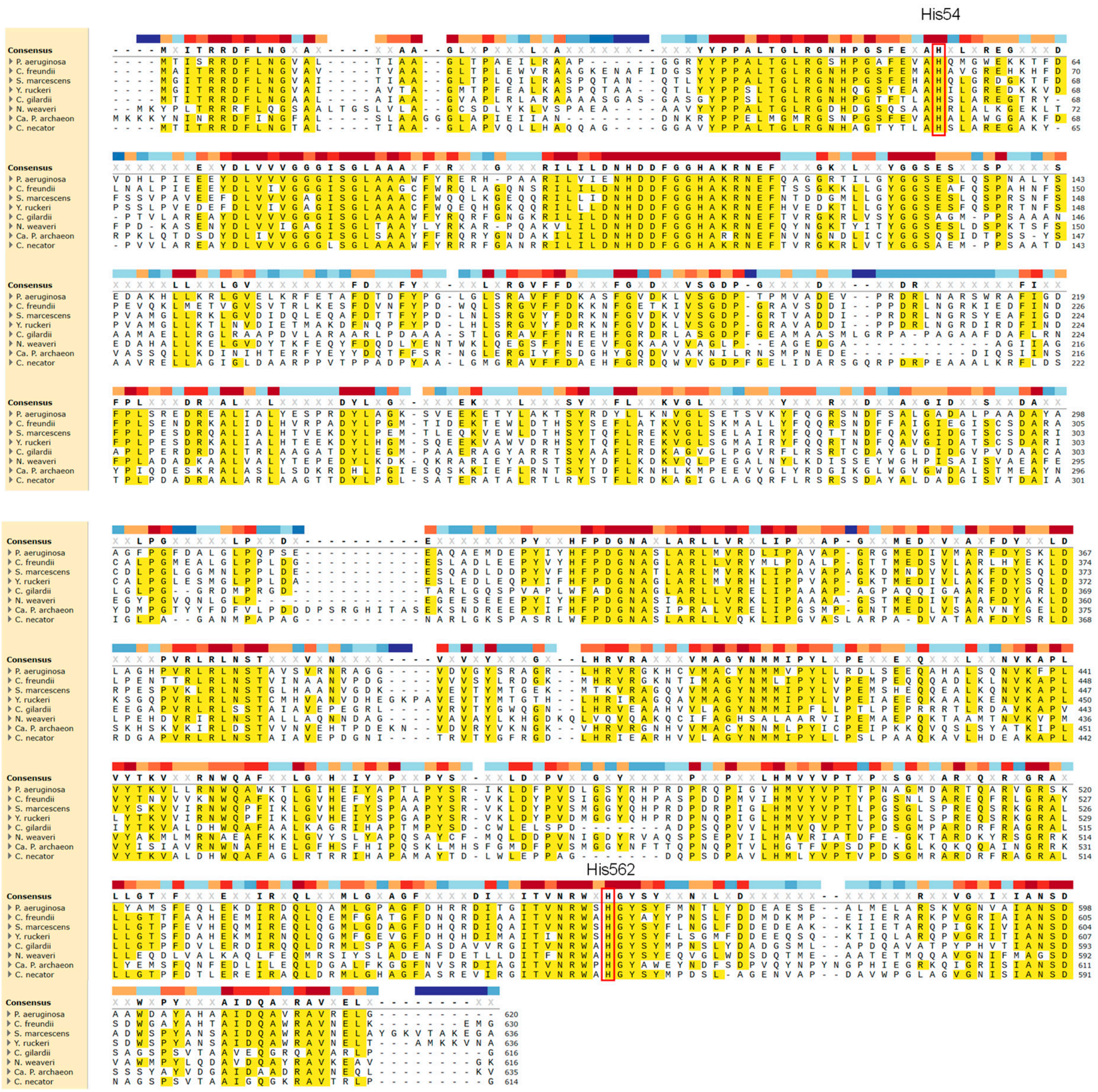

Figure S8. Entire primary structure alignment of PaSpdH and its homologs from seven bacterial species: *Citrobacter freundii* (GenBank: BBV36094), *Serratia marcescens*, (GenBank: KMU50083), *Yersinia ruckeri* (GenBank: OEU25204), *Cupriavidus gilardii* (GenBank: TWG83342), *Neisseria weaveri* (GenBank: VEJ51186.1), *Candidatus Poseidoniales archaeon* (GenBank: DAC18916), and *Cupriavidus necator* (GenBank: WP_042885275).

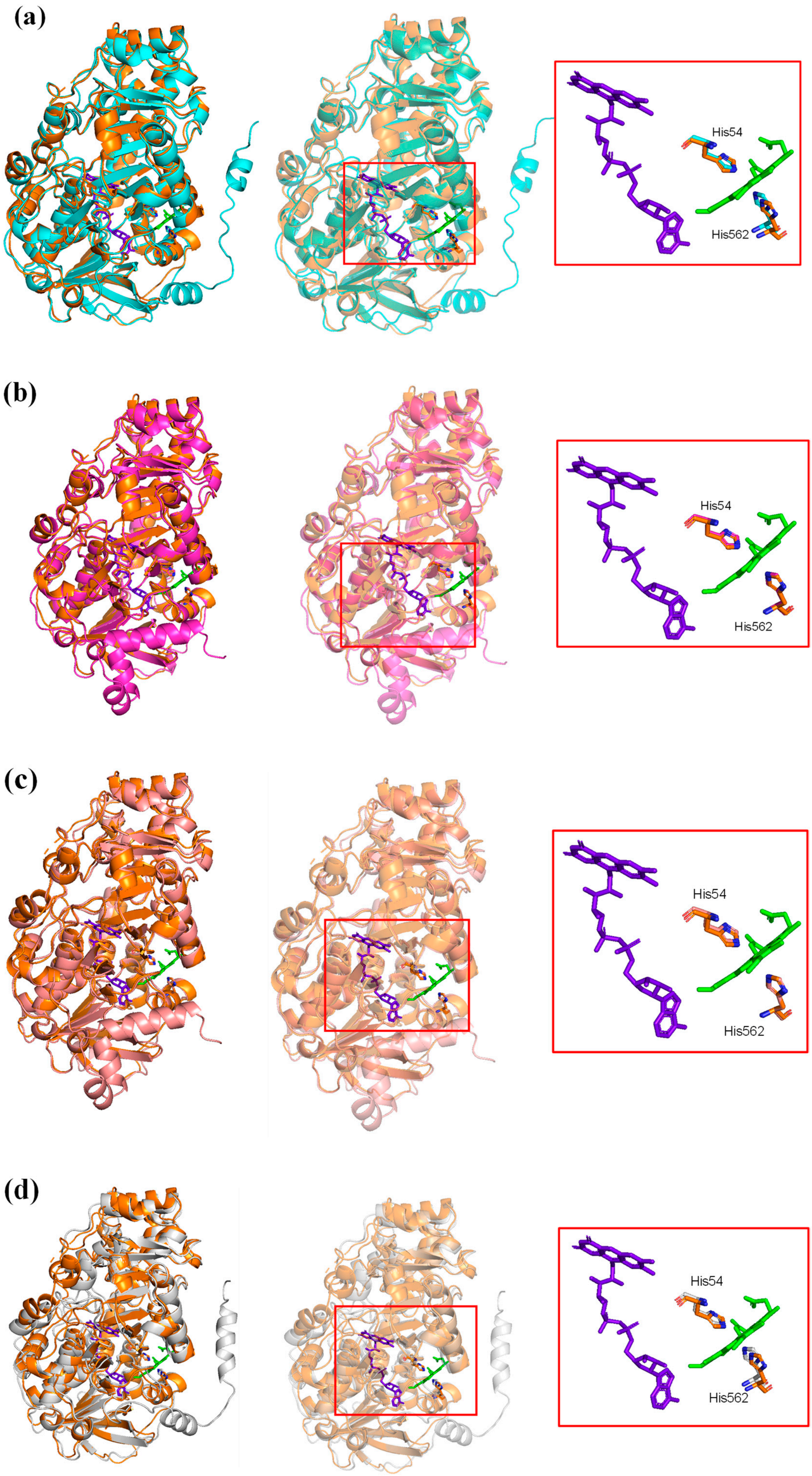
(a)
His54
His562
(b)
His54
His562
(c)
His54
His562
(d)
His54
His562

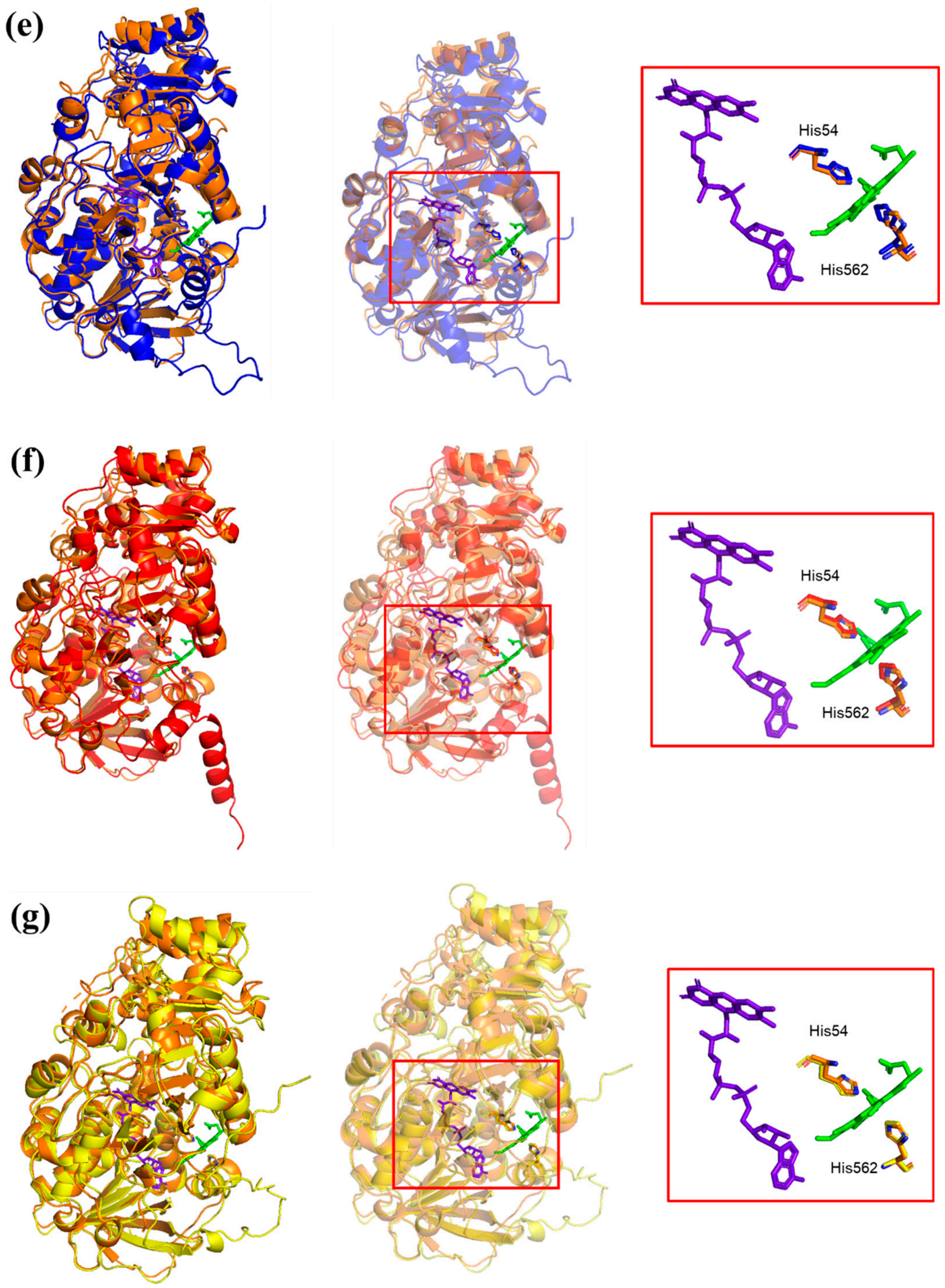

Figure S9. Structure of PaSpdH and its homologs predicted by AlphaFold3.

Orange: *P. aeruginosa* (PDB ID: 7D9G), purple: FAD, green: heme b, (a) Cyane: *Citrobacter freundii* (*C. freundii*); (b) Magenta: *Serratia marcescens* (*S. marcescens*); (c) Pink: *Yersinia ruckeri* (*Y. ruckeri*); (d) White: *Cupriavidus gilardii* J11 (*C. gilardii*); (e) Blue: *Neisseria weaver* (*N. weaveri*); (f) Red: *Cupriavidus necator* (*C. necator*); (g) Yellow: *Candidatus Poseidoniales archaeon* (*C. Poseidoniales. archaeon*)
